# Quantitative real-time measurement of endothelin-1-induced contraction in single non-activated hepatic stellate cells

**DOI:** 10.1371/journal.pone.0255656

**Published:** 2021-08-03

**Authors:** Naoki Dohi, Momoka Yamaguchi, Reina Hase, Ryosuke Suzuki, Yumeto Wakabayashi, Ryota Nishiyama, Shin-ya Saito, Tomohisa Ishikawa

**Affiliations:** 1 Department of Pharmacology, School of Pharmaceutical Sciences, University of Shizuoka, Suruga Ward, Shizuoka City, Shizuoka, Japan; 2 Faculty of Veterinary Medicine, Okayama University of Science, Imabari City, Ehime, Japan; Cinvestav-IPN, MEXICO

## Abstract

Although quiescent hepatic stellate cells (HSCs) have been suggested to regulate hepatic blood flow, there is no direct evidence that quiescent HSCs display contractile abilities. Here, we developed a new method to quantitatively measure the contraction of single isolated HSCs and evaluated whether endothelin-1 (ET-1) induced contraction of HSCs in a non-activated state. HSCs isolated from mice were seeded on collagen gel containing fluorescent beads. The beads around a single HSC were observed gravitating toward the cell upon contraction. By recording the movement of each bead by fluorescent microscopy, the real-time contraction of HSCs was quantitatively evaluated. ET-1 induced a slow contraction of non-activated HSCs, which was inhibited by the non-muscle myosin II inhibitor blebbistatin, the calmodulin inhibitor W-7, and the ET_A_ receptor antagonist ambrisentan. ET-1-induced contraction was also largely reduced in Ca^2+^-free conditions, but sustained contraction still remained. The tonic contraction was further diminished by the Rho-kinase inhibitor H-1152. The mRNA expression of P/Q-type voltage-dependent Ca^2+^ channels (VDCC), as well as *STIM* and *Orai*, constituents of store-operated channels (SOCs), was observed in mouse non-activated HSCs. ET-1-induced contraction was not affected by amlodipine, a VDCC blocker, whereas it was partly reduced by Gd^3+^ and amiloride, non-selective cation channel blockers. However, neither YM-58483 nor SKF-96365, which inhibit SOCs, had any effects on the contraction. These results suggest that ET-1 leads to Ca^2+^-influx through cation channels other than SOCs and produces myosin II-mediated contraction of non-activated HSCs via ET_A_ receptors, as well as via mechanisms involving Ca^2+^-calmodulin and Rho kinase.

## Introduction

Hepatic stellate cells (HSCs), located in the space of Disse between sinusoidal endothelial cells and hepatocytes, constitute 5%–8% of the total number of liver-resident cells [1]. Under physiological conditions, HSCs exist in a quiescent state, called quiescent HSCs (qHSCs), accumulating lipid droplets containing vitamin A [2]. Upon liver injury by alcohol consumption or viral infection, qHSCs are activated and transdifferentiated into myofibroblast-like activated HSCs (aHSCs) that produce collagen and undergo enhanced cell proliferation. Accordingly, the activation of HSCs is likely a cause of liver fibrosis and cirrhosis. In addition, aHSCs express α-smooth muscle actin (α-SMA), smooth muscle myosin, and L-type voltage-dependent Ca^2+^ channel (VDCC) [3], thereupon exhibiting contractile properties [4]. aHSCs are also suggested to be a cause of portal hypertension [4]. In contrast, the contractile properties of qHSCs under physiological conditions are not clear.

qHSCs encircling the sinusoid are anatomically similar to pericytes in capillaries. Therefore, qHSCs are postulated to participate in the regulation of blood flow in the liver [5]. Moreover, the contraction of rat qHSCs has been suggested to contribute to sinusoidal resistance [6]. Interestingly, endothelin-1 (ET-1), which was originally identified as a 21 residue potent vasoconstrictor peptide [7], has been shown to decrease sinusoidal diameter and increase sinusoidal resistance in perfused rat livers [8]. Isolated rat HSCs have been shown to display contractile activity in response to ET-1 [9]. The contraction of human HSCs has also been suggested to be induced by a chemokine CXCL12 through a calcium-independent pathway [10]. In these studies, contraction was evaluated by observing the shrinkage of collagen lattices on which isolated HSCs were cultured. Since isolated qHSCs are well known to be easily activated by culture on plastic plates [14], the preparations used in these studies might have contained aHSCs when the measurement was performed. In addition, since the shrinkage of collagen lattices develops very slowly, the responses to reagents were measured more than 24 h after their application. It is thus difficult to properly evaluate contraction of qHSCs without denying the involvement of aHSCs with this method.

In the present study, we have developed a novel method to quantitatively evaluate contractile properties in single isolated non-activated HSCs in real-time. The results shown here provide direct evidence for ET-1-induced contraction of non-activated HSCs.

## Materials and methods

### Isolation of HSCs from mice

**Fig 1:**
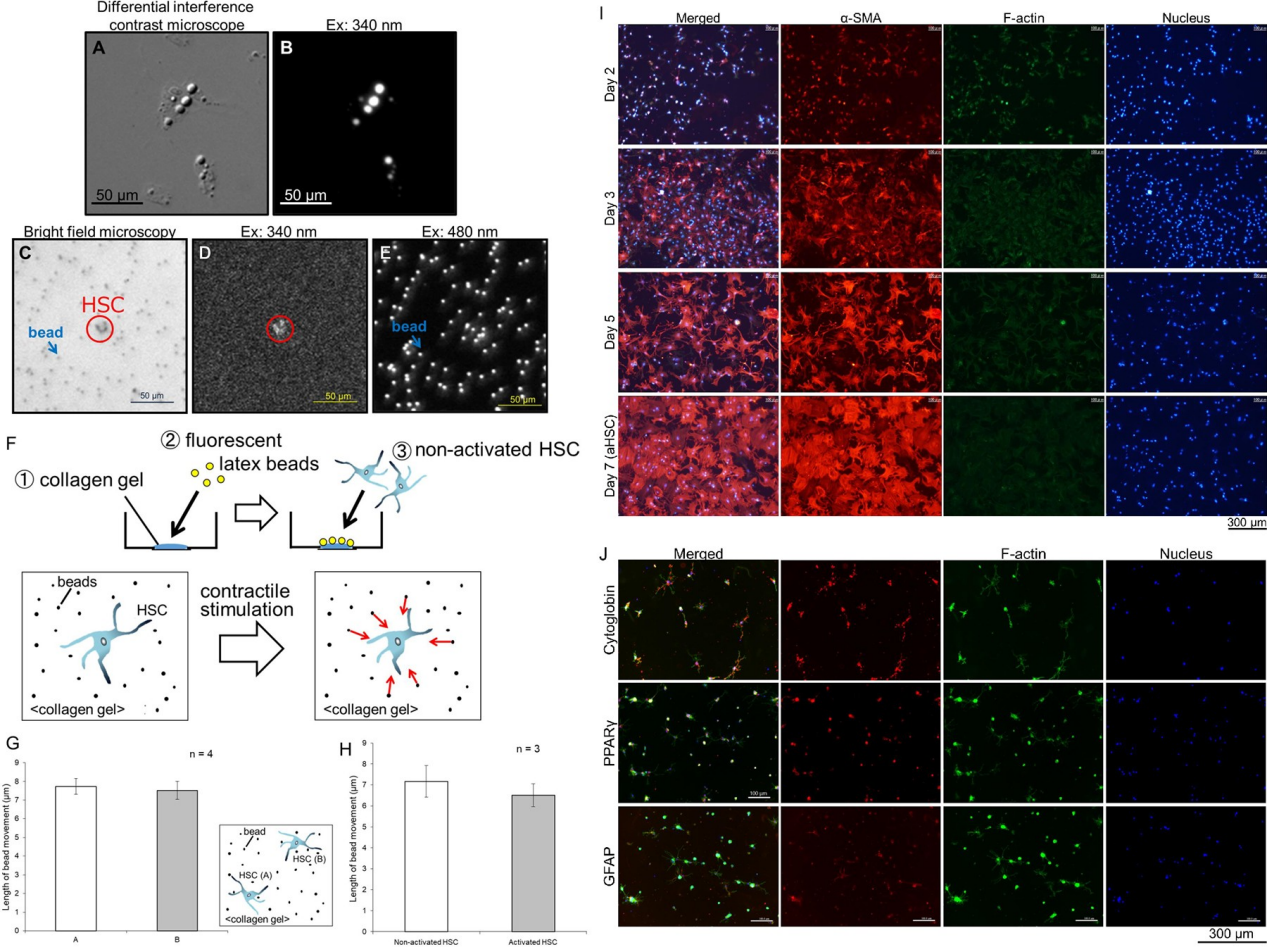
Method used to measure contraction of single non-activated hepatic stellate cells. (A) Differential interference contrast and (B) vitamin A autofluorescence (excited at 340 nm and emitted at 550 nm) images of isolated mouse non-activated HSCs cultured on collagen gel for less than 24 h. Arrows indicate oil-droplets. (C) Phase contrast, (D) vitamin A autofluorescence in non-activated HSCs (excited at 340 nm and emitted at 550 nm), and (E) latex bead-fluorescence (excited at 480 nm and emitted at 550 nm) images just before measurement. Arrows and circles indicate beads and non-activated HSCs, respectively. (F) Scheme of the measuring procedure. Isolated mouse non-activated HSCs were cultured on collagen gel containing 2.0-μm fluorescent latex beads (λ_ex_ = 470 nm, λ_em_ = 505 nm) in 35-mm glass bottom dishes. The movement of fluorescent latex beads was measured as an index of the contraction of non-activated HSCs. (G) Comparison of 10-nM ET-1-induced contraction between two non-activated HSCs, which were more than 350 μm apart from each other in the same viewing field. (H) Comparison of 10-nM ET-1-induced contraction between non-activated and activated HSCs. (I) Immunostaining images with anti-α-smooth muscle actin (α-SMA) antibody (red) of HSCs cultured for 2, 3, 5, and 7 days in DMEM containing 10% FBS on plastic plates. Nuclei and F-actin were stained with Hoechst 33342 (blue) and Alexa Fluor 488 phalloidin (green), respectively. (J) Immunostaining images with anti-cytoglobin, anti-PPARγ or anti-GFAP antibody (red) of HSCs cultured for 24 h in DMEM containing 10% FBS on collagen gel. Nuclei and F-actin were stained with Hoechst 33342 (blue) and Alexa Fluor 488 phalloidin (green), respectively.

Male ddY mice (8–12 weeks old; Nihon SLC, Hamamatsu, Japan) were housed under a 12 h light-dark cycle, with food and water available *ad libitum* and treated as approved by the Institutional Animal Care and Use Committee of the University of Shizuoka and in accordance with the Guidelines for Animal Experiments established by the Japanese Pharmacological Society. HSCs were isolated from mice anesthetized with isoflurane via digestion with pronase (Merck-Millipore, Tokyo, Japan), DNase I (Roche, Basel, Switzerland), and collagenase (Yakult, Tokyo, Japan), followed by density gradient centrifugation with 13% Histodenz (Sigma Aldrich, St. Louis, MO, USA) as described previously [11]. One-day and 7-days primary cultured HSCs were used as non-activated and activated cells, respectively. Cells immunostained with antibody against α-SMA, a marker of aHSC, was markedly increased when cultured in DMEM containing FBS on plastic plates for 7 days (Fig 1I).

### Measurement of contraction

The contraction of isolated HSCs was measured on a solid gel mixture containing 2.0-μm fluorescent latex beads (Sigma Aldrich, #L4530-1ML). The gel mixture was made with rat tail type I collagen (Millipore, Darmstadt, Germany) and 2.65× Dulbecco’s modified Eagles medium (DMEM, Nissui Pharmaceutical, Tokyo, Japan) and adjusted to pH 7 with 0.5 N NaOH. 150 μL of the gel mixture was plated on a 35 mm glass bottom dish (glass diameter, 14 mm; Matsunami Glass, Osaka, Japan) and flattened. The fluorescent latex beads were diluted 4000-fold with MilliQ water and sonicated for 1 min, and 400 μL of the solution was placed on the gel. The final concentration of collagen in the dish was 1 mg/mL. Collagen was then solidified in a 5% CO_2_ incubator at 37°C. Isolated HSCs, which were cultured in DMEM supplemented with 10% fetal bovine serum (Biowest, Cat No: S1820-500, Lot No: S08048S1820), 100 units/mL penicillin, and 100 mg/mL streptomycin and incubated at 37°C in a humidified atmosphere with 5% CO_2_ for less than 24 h, were seeded on the solid gel mixture 4 h after the gel preparation. For contraction measurement, the dish solution was replaced by HEPES-buffered solution (125 mM NaCl, 5 mM KCl, 1.3 mM MgSO_4_·7H_2_O, 1.2 mM CaCl_2_·2H_2_O, 20 mM HEPES, 5.8 mM D-glucose, pH 7.2–7.4).

### Measurement of mRNA expression

Total RNA was extracted from non-activated and activated HSCs using the RNeasy Mini kit according to the manufacturer’s instructions (Qiagen, Tokyo, Japan). RT-PCR was performed using Qiagen One Step RT-PCR kit (Qiagen, Tokyo, Japan). Primers used for RT-PCR are listed in Table 1.

**Table 1:** Primers used for RT-PCR.

	Forward	Reverse
GAPDH	AACGACCCCTTCATTGAC	TCCACGACATACTCAGCAC
MLCK	CCAATCAATGCGGAGAAACT	CAGGGTCTGGGTATCCTTCA
MYPT1	AAGCACCACATCAACACCAA	GAGCCACAGGAATGGTCACT
NMMIIa	AGGAGACAAAGGCGCTATCA	GACTTCTCCAGCTCGTGGAC
NMMIIb	AAACAAACTCCAGGCCCTTT	AGACCAGGTGTGGTTGAAGG
ET_A_	TCCGAGGAGCTCTAAGGTGA	GTGGTGCCCAGAAAGTTGAT
ET_B_	TTCACTCCCCAGTTGGTCTC	GTCTTAGTGGGTGGCGTCAT
T-α_1G_	TCATAGCCGTGCTGATGAAG	AAGGGAGAAGCCTGAAGAGG
T-α_1H_	CCTTTCTCAGCGTCTCCAAC	GCCACAATGATGTCAACCAG
T-α_1I_	CATGAAGACCATGGACAACG	ACAGGACAACGTTCCAGTCC
R-α_1E_	CAGCTCCCTGATGAGACACA	ATGTAGTGCTGTGGGGAAGG
L-α_1S_	GACAGCAGAGGAGGAACTGG	AGCAAATTGGAGGGGTCTTT
L-α_1C_	GCATCACAATCAGCAGGCTA	ACGGGGTTCTACAGGCTTTT
L-α_1D_	TGCACAGATGAAGCCAAAAG	GACCAACGTTCTCACCGTTT
L-α_1F_	GGAGGATGTCGGAATCTGAA	GTTTGTTGTGCTGGGTCCTT
P/Q-α_1A_	CTGCTTTGAAGAGGGGACAG	GGAAAACAGTGAGCACAGCA
STIM1	GGTAGCCGAAACACACGAAT	GAAAGGAAGGGAGGTGAAGG
STIM2	GACACGCCCACCTCATAACT	TTTTCCGTTCCAGATTTTGC
Orai1	TCCCTGGTCAGCCATAAGAC	TCATGGAGAAGGGCATAAGG
Orai2	GGGGCCAGTACTTACCCATT	GCTTGCCAGTATGACCCATT
Orai3	GCCACCTCCTGTAAGCTCTG	TCCTGGAGGAGCAAACAACT
TRPC1	AGCCTCTTGACAAACGAGGA	TCTTACAGGTGGGCTTACGG
TRPC2	GCCAGCAAGTTCTGTCTTCC	ACCAGAGACTCTCCCAGCAA
TRPC3	ATTCTTCGAAGCCCCTTCAT	ACGTGAACTGGGTGGTCTTC
TRPC4	GCTGGAGGAGAAGACACTGG	GACCTGTCGATGTGCTGAGA
TRPC5	GGAGGCACAACTTGAGAAGC	TGGAGAGGCTTCTTCTTGGA
TRPC6	GCAAGGATTTCGTTGTTGGT	TGCTGACAGTTTGGATGAGC
TRPC7	TCCTGGGCACATGACTGATA	AGAGCTGACTTCCCAGGACA
TRPV1	AGCGAGTTCAAAGACCCAGA	TTCTCCACCAAGAGGGTCAC
TRPV2	TGATGAAGGCTGTGCTGAAC	CACCACAGGCTCCTCTTCTC
TRPV4	ACAACACCCGAGAGAACACC	TGAACTTGCGAGACAGATGC
TRPM4	CAGCGACCTCTACTGGAAGG	CACAGACTCCCAAGTCAGCA
TRPM5	GGAACGACCTTTGGCTATGA	GCCACTACACGGATCTTGGT
TRPM7	TTTGGTGTTCCCAGAAAAGC	ACCAAGTTCCAGGACCACAG
TRPA1	ATATGCAGTGGCAATGTGGA	CTGAGGCCAAAAGCCAGTAG

Notes. GAPDH: glyceraldehyde-3-phosphate dehydrogenase, ET_A_, ET_B_: Endothelin receptor type A, B, T-α1G: T-type voltage-dependent calcium channel (VDCC) α1G subunit, T-α1H: T-type VDCC α1H subunit, T-α1I: T-type VDCC α1I subunit, R-α1E: R-type VDCC α1E subunit, L-α1S: L-type VDCC α1S subunit, L-α1C: L-type VDCC α1C subunit, L-α1D: L-type VDCC α1D subunit, L-α1F: L-type VDCC α1F subunit, P/Q-α1A: P/Q-type VDCC α1A subunit, STIM 1, 2: stromal interaction molecule 1, 2, Orai 1–3: calcium release-activated calcium modulator 1–3, TRPC1-7: transient receptor potential (TRP) cation channel subfamily C member 1–7, TRPV1, 2, 4: TRP cation channel subfamily V member 1, 2, 4, TRPM4, 5, 7: TRP cation channel subfamily M member 4, 5, 7, TRPA1: TRP cation channel subfamily A member 1.

### Immunostaining

Cells cultured on 96-well plates were fixed with 2% paraformaldehyde (Wako, Osaka, Japan) for 45 min, permeabilized with 0.1% Triton X-100 for 1 h, and blocked with 3% bovine serum albumin fraction V (BSA, Roche, Mannheim, Germany) for 30 min in phosphate-buffered saline (PBS; 137 mM NaCl, 8.10 mM Na2HPO4∙12H2O, 2.68 mM KCl, 1.47 mM KH2PO4). The cells were then incubated with mouse monoclonal anti-α-smooth muscle actin (α-SMA) antibody (1:1000, Sigma, St. Louis, MO, USA, #A2547), anti-cytoglobin polyclonal antibody produced in rabbit (1;750, Cloud-clone, Katy, TX, USA, #PAC426Ra01), anti-peroxisome proliferator-activated receptor γ (PPARγ) polyclonal antibody produced in rabbit (1:750, Cloud-clone #PAA886Mu01), or anti-glial fibrillary acidic protein (GFAP) antibody produced in rabbit (1:750, Cell Signaling Technology, Danvers, MA, USA #12389S), in PBS containing 5% BSA overnight at 4°C, followed by incubation with Alexa Fluor® 546 conjugated goat anti-mouse IgG antibody (1:750, Invitrogen, Carlsbad, CA, USA) or Alexa Fluor® 546 conjugated goat anti-rabbit IgG antibody (1:1000, Invitrogen), and Alexa Fluor 488 conjugated phalloidin (1:750, Invitrogen) in PBS containing 5% BSA for 1 h at room temperature. Nuclei were stained with Hoechst 33342 (1:1500, Dojindo, Kumamoto, Japan).

### Reagents

The following reagents were used: ambrisentan (BSF208075; Adooq Bioscience, Irvine, CA, USA); NOC7 (Dojindo, Kumamoto, Japan); collagen type I (rat tail) and PRONASE protease, *Streptomyces griseus* (Merck-Millipore); endothelin-1 (PEPTIDE Inst., Osaka, Japan); dantrolene sodium salt, H-1152 dihydrochloride, HistoDenz, ML-9, thapsigargin (Sigma Aldrich); BQ-788 sodium salt (Tocris Bioscience, Bristol, UK); collagenase-Yakult (Yakult, Tokyo, Japan); and W-7 and xestspongin C (Wako Pure Chemical, Osaka, Japan).

### Statistics

Data are expressed as means ± SEM. Groups were compared using the Holm–Bonferroni multiple comparison test or the unpaired Student’s *t*-test. *P* values < 0.05 was considered statistically significant.

## Results

### Measurement of HSC contraction induced by ET-1

Non-activated HSCs were identified by autofluorescence of vitamin A, which was excited at 340 nm and emitted at 550 nm (Fig 1A–1D). One HSC was selected, and the movement of fluorescent latex beads, which were excited at 470 nm and emitted at 505 nm, around this cell was measured as an index of HSC contraction (Fig 1E). Fluorescence images were recorded at a frequency of 0.2 Hz using the AQUACOSMOS system (Hamamatsu Photonics, Hamamatsu, Japan). The recorded images were loaded onto ImageJ software and 8–16 fluorescent beads, which were located between 35 and 150 μm from the cell, were selected for the contraction measurement. The x, y-coordinates of each bead in the continuous images were measured using Particle Track and Analysis plugins for ImageJ and outputted in a text file (Fig 1F). The length of bead movement was calculated from the changes in the coordinates (Fig 1F). The data were averaged and used as the index of cell contraction. Since beads located within approximately 250 μm from the cell moved associated with cell contraction induced by 10 nM ET-1 *vide infra*, the cell which was more than 350 μm apart from other cells was selected for the measurement to avoid interference between cells (Fig 1G). There was no difference in the contraction between two cells, which were apart more than 350 μm, in the same viewing field (Fig 1G). We also confirmed that the beads more than 400 μm apart from the cell did not move when the cell contracted (S2 Fig).

**Fig 2:**
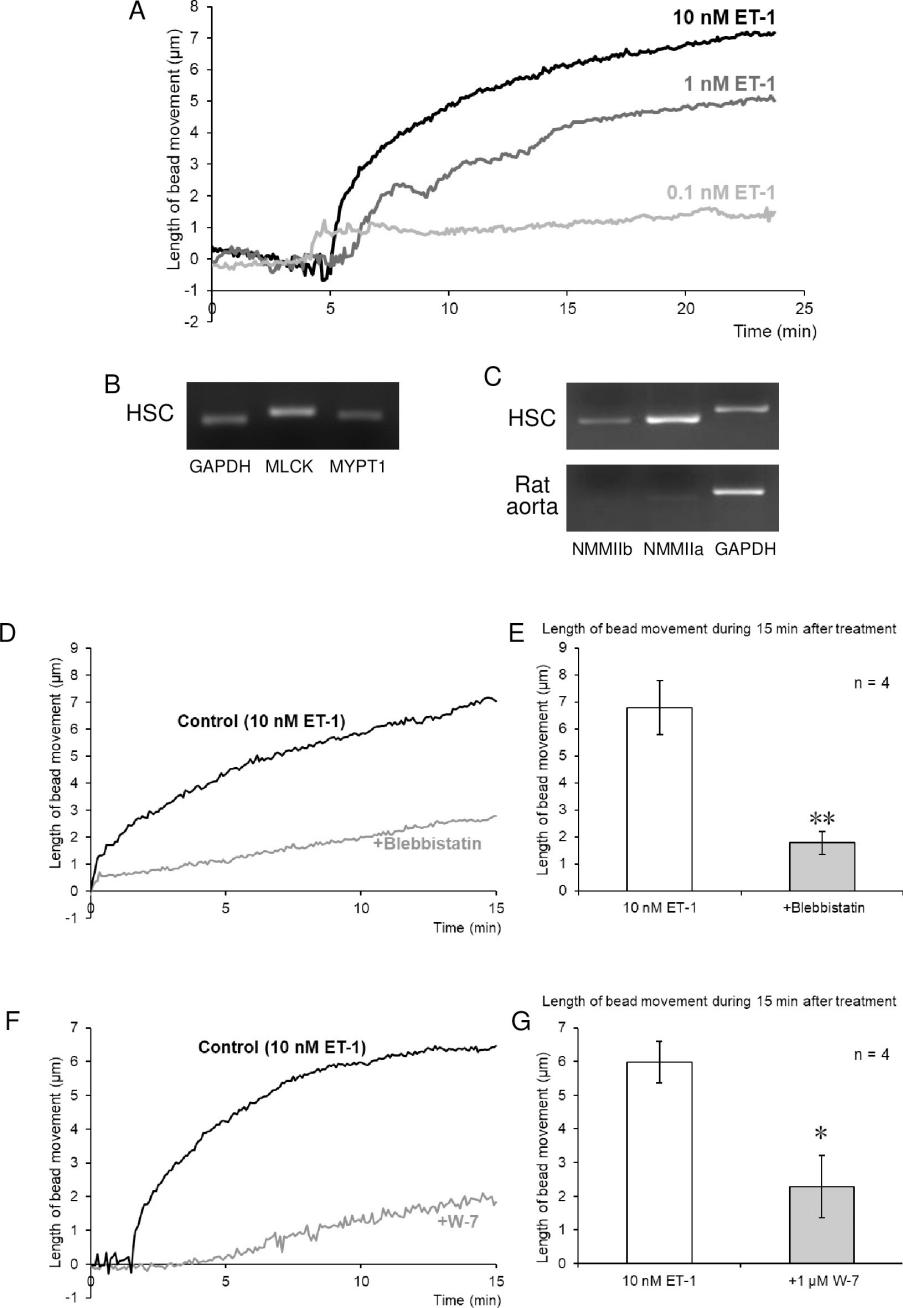
Endothelin-1 (ET-1)-induced contraction of isolated mouse non-activated HSCs. (A) Typical traces of non-activated HSC contraction. ET-1 induced concentration-dependent sustained contraction of non-activated HSCs. (B) RT-PCR analysis of mRNA expression of myosin light chain kinase (*MLCK*) and myosin phosphatase target subunit 1 (*MYPT1*) in non-activated HSCs. (C) RT-PCR analysis of mRNA expression of non-muscle myosin IIa and IIb (*NMM IIa*, *IIb*), and IIb in non-activated HSCs and rat aorta. Effects of the selective NMM inhibitor blebbistatin (D, E) and the calmodulin inhibitor W-7 (F, G) on ET-1-induced contraction. Traces represent a typical time-course of changes in the length of bead movement after the treatment with ET-1 (10 nM) in the absence and presence of blebbistatin (10 μM; D) or W-7 (1 μM; F). Traces in (A), (D), and (F) were obtained from different cells. Bar graphs indicate the maximal contraction within 15 min after treatment with ET-1 (10 nM) in the absence and presence of blebbistatin (10 μM; E) or W-7 (1 μM; G). Each bar represents the mean ± S.E.M. (n = 4). **P < 0.01, *P < 0.05.

Using the single-cell contraction analysis, we directly demonstrated that ET-1 (0.1, 1, and 10 nM) induced a sustained contraction of isolated mouse non-activated HSCs in a concentration-dependent manner (Fig 2A and S1 Video). The contraction developed slowly, reaching a maximum contraction at around 15 min. Similarly, ET-1 (10 nM) induced a sustained contraction of mouse aHSCs, which were obtained by cultivating for 7 days (Fig 1H). The culturing of isolated primary HSCs in DMEM containing FBS for 5 or 7 days on plastic plates was shown to lead to marked changes in cell shape and an increase in the area of α-SMA-positive staining, which is a marker of aHSC (Fig 1I). We further confirmed that isolated primary HSCs cultured in DMEM containing FBS for 24 h on the collagen gel were kept in a non-activated state, which was demonstrated by immunocytochemistry for cytoglobin, PPARγ, and GFAP, which are known as markers of qHSC (Fig 1J).

RT-PCR analysis showed the mRNA expression of myosin light chain kinase (*MLCK*) the myosin phosphatase targeting subunit (*MYPT1*), the regulatory subunit of myosin light chain phosphatase, and non-muscle myosin IIa (*NMM IIa*) and IIb (*NMM IIb*) in mouse non-activated HSCs (Fig 2B and 2C). We also confirmed that the contraction of non-activated HSCs induced by ET-1 (10 nM) was suppressed by blebbistatin (10 μM), an inhibitor of NMM II and striated muscle myosin II (Fig 2D and 2E), and by W-7 (1 μM), a calmodulin (CaM) inhibitor (Fig 2F and 2G).

### Mechanism of non-activated HSC contraction induced by ET-1

**Fig 3:**
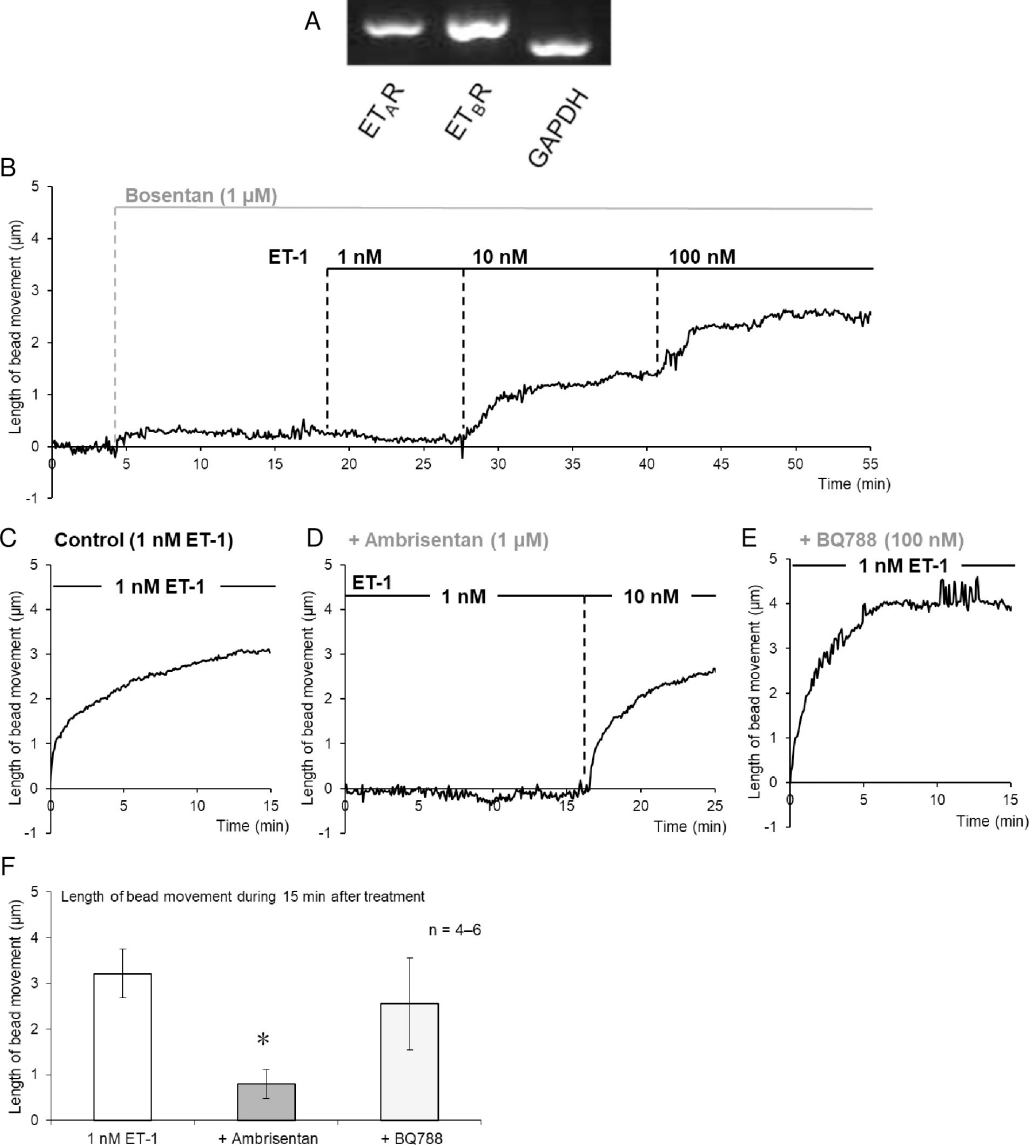
Involvement of ET_A_ receptors in ET-1-induced contraction of isolated mouse non-activated HSCs. (A) RT-PCR analysis of the expression of ET-1 receptors in non-activated HSCs. The mRNA expression of ET_A_ and ET_B_ receptors was detected in non-activated HSCs. (B–F) Effects of endothelin receptor antagonists on ET-1-induced contraction. Traces represent a typical time-course of changes in length of bead movement after treatment with ET-1 (1, 10 or 100 nM) in the absence (C; Control) or presence of bosentan (B; 1 μM, an antagonist of both ET_A_ and ET_B_ receptors), ambrisentan (D; 1 μM, a selective ET_A_ receptor antagonist) or BQ788 (E; 100 nM, a selective ET_B_ receptor antagonist). (F) Bar graphs indicate the maximal contraction within 15 min after treatment with ET-1 (1 nM) in the absence and presence of ambrisentan (1 μM) or BQ788 (100 nM). Each bar represents the mean ± S.E.M. (n = 4–6). *P < 0.05 vs. control (without antagonists).

The mRNA expression of both ET_A_ and ET_B_ receptors in non-activated HSCs was confirmed using RT-PCR (Fig 3A). Pharmacological analysis with ET receptor antagonists with different specificities was thus performed to determined which subtypes were involved in the observed contraction. Bosentan (1 μM), a non-selective ET-1 receptor antagonist, abolished the contraction induced by ET-1 (1 and 10 nM; Fig 3B). Ambrisentan (1 μM), a selective ET_A_ receptor antagonist, also significantly reduced the contraction induced by ET-1 (1 nM; Fig 3C, 3D and 3F). In contrast, BQ-788 (100 μM), a selective ET_B_ receptor antagonist, showed no significant effect on the contraction induced by ET-1 (1 nM; Fig 3C and 3E, 3F).

**Fig 4:**
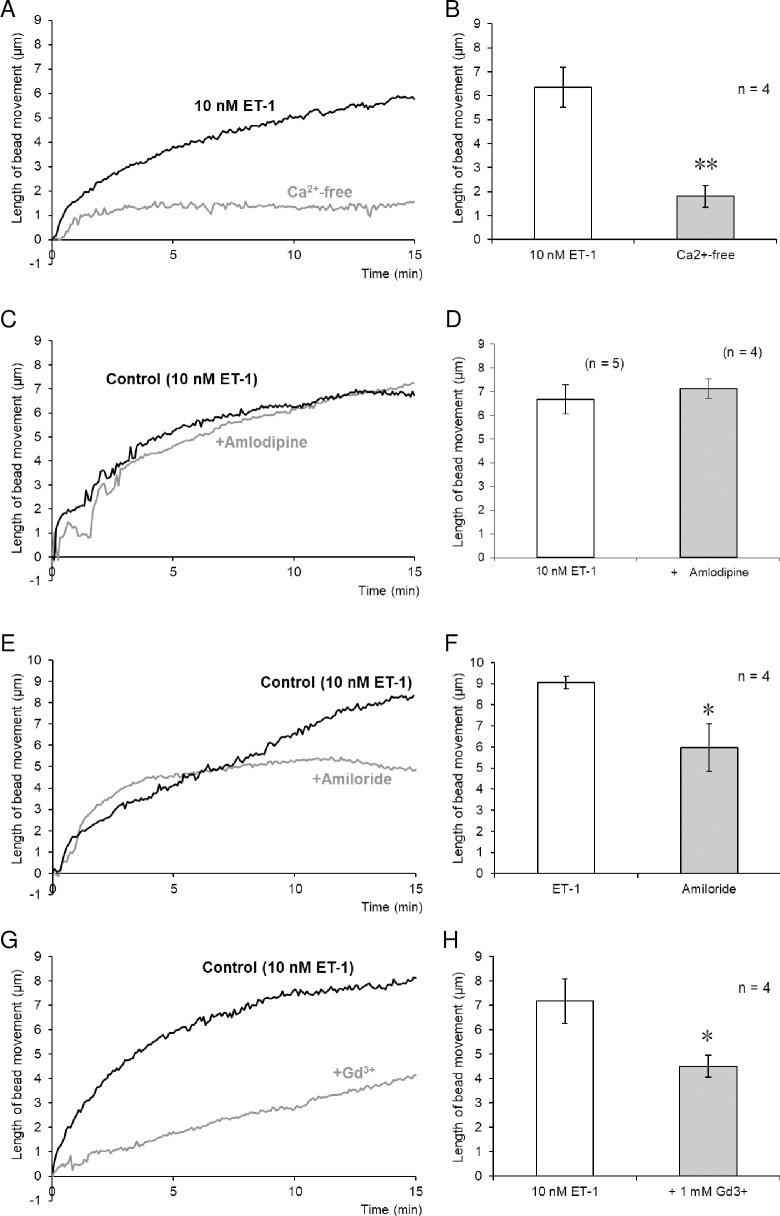
Involvement of Ca^2+^ influx in ET-1-induced contraction of isolated mouse non-activated HSCs. (A–H) Effects of extracellular Ca^2+^ removal, a Ca^2+^ channel blocker, and cation channel blockers on ET-1-induced contraction. Traces represent typical time-courses of changes in length of bead movement after treatment with ET-1 (10 nM) in control and Ca^2+^-free solution containing 1.5-mM EGTA (A), or in the absence and presence of amlodipine (C; 1 μM, an L- and P/Q-type Ca^2+^ channel blocker), amiloride (E; 100 μM, a cation channel blocker), or Gd^3+^ (G; 1 mM, a cation channel blocker). Traces in (A), (C), (E), and (G) were obtained from different cells. Bar graphs indicate the maximal contraction within 15 min after treatment with ET-1 (10 nM) in control and Ca^2+^-free solution containing 1.5-mM EGTA (B), or in the absence and presence of amlodipine (D; 1 μM), amiloride (F; 100 μM), or Gd^3+^ (H; 1 mM). Each bar represents the mean ± S.E.M. (n = 4–5). **P < 0.01, *P < 0.05.

RT-PCR analysis also showed the mRNA expression of P/Q-type VDCC, as well as *STIM* and *Orai*, constituents of store-operated channels (SOCs), in mouse non-activated HSCs and aHSCs, although no apparent difference was observed between the cells (S3 Fig). We therefore investigated whether ET-1-induced contraction of non-activated HSCs is mediated by P/Q-type VDCCs. Amlodipine (1 μM), which inhibits not only L-type but also P/Q-type VDCCs [12], had no effect on non-activated HSC contraction induced by ET-1 (10 nM; Fig 4C and 4D). In contrast, non-activated HSC contraction induced by ET-1 (10 nM) was largely reduced in Ca^2+^-free solution containing EGTA (1.5 mM; Fig 4A and 4B). Moreover, Gd^3+^ (1 mM), a non-selective cation channel blocker, largely reduced ET-1-induced non-activated HSC contraction (Fig 4G and 4H). Amiloride (1 μM), another non-selective cation channel blocker, preferentially reduced the sustained phase of ET-1-induced contraction (Fig 4E and 4F).

**Fig 5:**
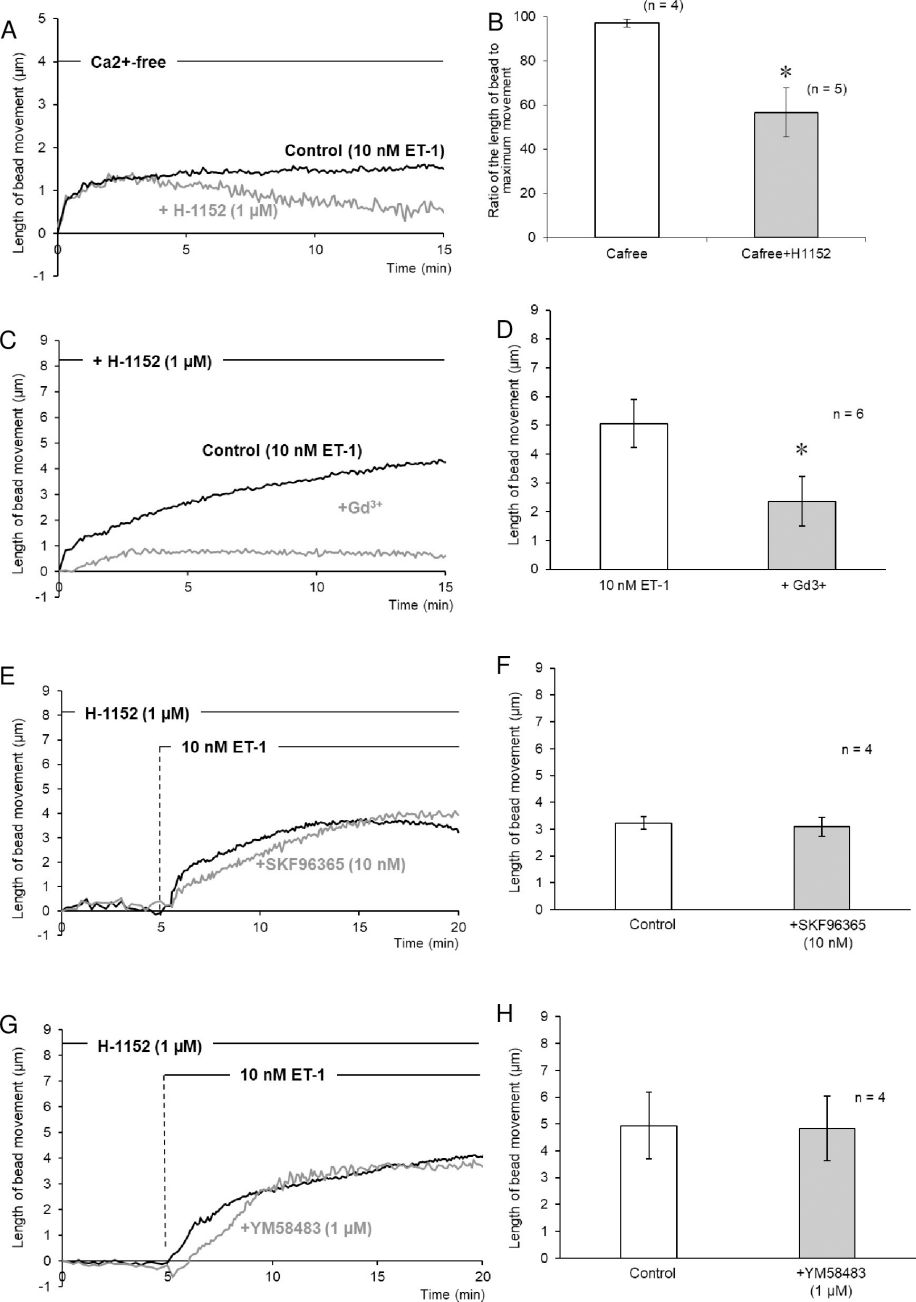
Ca^2+^ influx-independent ET-1-induced contraction of isolated mouse non-activated HSCs. (A, B) Effect of the Rho-kinase inhibitor H-1152 on ET-1-induced contraction in Ca^2+^-free solution containing 1.5-mM EGTA. (A) Traces represent typical time-courses of changes in length of bead movement after treatment with ET-1 (10 nM) in the absence and presence of H-1152 (1 μM). (B) Bar graphs represent contraction 15 min after treatment with ET-1 (10 nM) in the absence and presence of H-1152 (1 μM). (C–H) Effects of cation channel inhibitors on ET-1-induced contraction in the presence of H-1152 (1 μM). Traces represent typical time-courses of changes in length of bead movement after treatment with ET-1 (10 nM) in the absence and presence of Gd^3+^ (C; 1 mM, a cation channel blocker), SKF96365 (E; 10 nM, a TRP channel blocker), or YM58483 (G; 1 μM, an SOC blocker). Bar graphs indicate the maximal contraction 15 min after treatment with ET-1 (10 nM) in the absence and presence of Gd^3+^ (D; 1 mM), SKF96365 (F; 10 nM), or YM58483 (H; 1 μM). Each bar represents the mean ± S.E.M. (n = 4–6). *P < 0.05.

We then further investigated the remaining sustained contraction induced by ET-1 in Ca^2+^-free conditions. In the presence of H-1152 (1 μM), a Rho-kinase inhibitor, in Ca^2+^-free solution containing EGTA (1.5 mM), the contraction induced by ET-1 (10 nM) was not sustained, but rather gradually decreased (Fig 5A). The contraction at 15 min after treatment with ET-1 (10 nM) was significantly smaller in the presence of H-1152 (1 μM; Fig 5B). Since the involvement of Rho kinase in ET-1-induced contraction was suggested, further experiments were performed in the presence of H-1152. ET-1-induced contraction in the presence of H-1152 (1 μM) was largely inhibited by Gd^3+^ (1 mM; Fig 5C and 5D). In contrast, YM-58483 (1 μM); Fig 5E and 5F), an SOC blocker, or SKF-96365 (10 μM; Fig 5G and 5H), a TRP channel blocker, had no effect on ET-1-induced contraction in the presence of H-1152 (1 μM). In addition, the combination of H-1152 (1 μM) and W-7(1 μM) caused complete relaxation of ET-1-induced contraction, whereas the NO donor NOC7 partially reversed it (S1 Fig).

## Discussion

Conventionally, the contraction of HSCs has been measured via shrinkage of collagen lattices containing isolated HSCs [9, 10, 13]. However, isolated qHSCs are well known to be easily activated by culture on plastic plates [14]; therefore, the involvement of aHSCs in the response is hard to be ruled out when using this method. In addition, this method to measure the shrinkage of collagen lattices was not suitable for measuring immediate contraction. In the present study, we have developed a novel method to measure the contraction of single primary non-activated HSCs cultured on collagen gel. Our results in immunostaining for qHSC markers, i.e., cytoglobin, PPARγ, and GFAP, suggest that HSCs seemed to be kept in a non-activated state by culturing on collagen gel used in the present study at least within 24 h after isolation. Due to the short culture time and the conditions of culture on collagen gel, the activation of HSCs might not be high. Since the activation of HSCs has been shown to revert by culture on soft gel [14, 15], the culture on the soft collagen gel, which was used in order to be able to monitor changes in cell length, might also have advantage of keeping HSCs in a non-activated state. Even if there exists aHSC in the preparation, the current method would enable to analyze contractility of single non-activated HSC without any interference by selecting the cell being more than 350 μm apart from other cells. In addition, the current method allows to measure contraction and relaxation in real-time and in immediate response to reagent treatment. We directly demonstrated that ET-1 induces a sustained contraction of non-activated HSCs, which is suggested to be mediated via ET_A_ receptors.

The present study showed the mRNA expression of NMM IIa and IIb in mouse non-activated HSCs and the inhibition of ET-1-induced non-activated HSC contraction by the myosin II inhibitor blebbistatin. These results suggested that non-activated HSC contraction induced by ET-1 is mediated by NMM II. This is the first report suggesting the involvement of NMM II in the contraction of non-activated HSCs. Although aHSCs are known to have contractile activity, NMM II has been suggested not to be involved in ET-1-induced aHSC contraction, as no apparent changes in ET-1-induced contraction of aHSCs were observed in rat aHSCs after knockdown of NMM IIa or IIb [16]. However, in that study, aHSC contraction was evaluated by changes in aHSC-seeded collagen lattice, which was measured 24 h after treatment with ET-1. The method developed in the present study would enable one to measure real-time changes in contraction even in aHSCs and provide more direct evidence for the mechanism of aHSC contraction.

ET-1-induced non-activated HSC contraction was largely dependent on extracellular Ca^2+^. Although the expression of P/Q-type VDCCs in non-activated HSCs was confirmed, the contribution of VDCCs to contraction was determined to be unlikely, since amlodipine, which blocks not only L-type but also P/Q-type VDCC, showed no inhibitory effect on ET-1-induced contraction. Other mediators of Ca^2+^ influx include cation channels, such as SOCs. The involvement of SOCs in ET-1-induced contraction was also determined to be unlikely, as SOC blockers SKF-96365 [17] and YM-58483 [18] had little effect on this contraction. In contrast, Gd^3+^, which blocks not only SOC at low concentrations, but also non-capacitative Ca^2+^ channels at high concentrations, largely reduced ET-1-induced contraction, indicating that cation channels other than SOCs are likely involved in the response. The involvement of cation channels was also supported by the results with amiloride, a broad-spectrum cation channel blocker.

In extracellular Ca^2+^-free conditions, ET-1 still induced contraction of non-activated HSCs. Ca^2+^-independent contraction induced by ET-1 has also been reported in rat aHSCs [19]. Since ET-1induced contraction in Ca^2+^-free solution, especially tonic contraction, was suppressed by the Rho-kinase inhibitor H-1152, we hypothesized the involvement of Rho kinase in Ca^2+^-independent tonic contraction. In rat HSCs, ET-1-induced contraction has been shown to be inhibited by Y-27632, a Rho-kinase inhibitor [20]. In this study, the formation of F-actin stress fibers disappeared in HSCs treated with Y-27632, suggesting that the inhibition of Rho kinase attenuates actin/myosin interaction in HSCs, resulting in impaired contraction. In the present study, however, no morphological changes in non-activated HSCs were observed, perhaps due to the short duration of treatment with H-1152. Alternatively, H-1152 might inhibit a Ca^2+^-sensitization mechanism. Rho kinase mediates Ca^2+^ sensitization in smooth muscle cells; it phosphorylates MYPT, a regulatory subunit of myosin phosphatase, resulting in the inhibition of myosin phosphatase [21–23]. Ca^2+^ sensitization is well known to be involved in ET-1-induced contraction of smooth muscle [24]. We confirmed the expression of *MYPT* mRNA in non-activated HSCs. Further experiments will be required to determine whether a Ca^2+^-sensitization mechanism mediated by the Rho/Rho kinase pathway is involved in ET-1-induced contraction of non-activated HSCs.

Endothelin receptors are classified into two subtypes, i.e., ET_A_ and ET_B_ receptors. It has been reported that in normal rats, mRNA expression of ET_B_ receptors is detected not only in sinusoidal endothelial cells, Kupffer cells, and hepatocytes, but also in qHSCs. mRNA expression of ET_A_ receptors, however, is detected in qHSC to a larger degree than in these other cell types [25]. The present pharmacological analysis using ET receptor antagonists with different specificity suggested that ET-1-induced contraction of non-activated HSCs is mediated primarily via ET_A_ receptors. However, unlike the non-selective ET receptor antagonist bosentan, the specific ET_A_ receptor antagonist ambrisentan did not completely inhibit ET-1-induced non-activated HSC contraction. Thus, the possibility that ET-1-induced non-activated HSC contraction is partly mediated via ET_B_ receptors could not be ruled out, although the selective ET_B_ receptor antagonist BQ-788 had no effect on the contraction. ET_A_ and ET_B_ receptor cross-talk has been shown to occur in ET-1-induced vascular contraction, which might explain the limited efficacy of selective ET_A_ or ET_B_ receptor antagonists compared to non-selective ET receptor antagonists [26, 27].

Under physiologically relevant conditions, liver ET-1 levels may be as low as 0.05 pg/mg tissue [28]. The plasma ET-1 level in patients with liver disease has been shown to be higher than that in healthy subjects; therefore, the role of ET-1 in the liver has been discussed in contribution to the development and progression of liver diseases such as portal hypertension [28]. However, since ET-1 is mainly synthesized in and released from sinusoidal endothelial cells that neighbor HSCs in the liver, it may affect HSCs through paracrine effects [29, 30]. Thus, the local concentration of ET-1 surrounding qHSCs could reach high enough to induce qHSC contraction even under physiological conditions where the plasma ET-1 level is low. We propose a pivotal role for qHSCs in the regulation of hepatic sinusoidal blood flow via their contractile activity under physiological conditions, potentially mediated by ET-1.

## Supporting information

S1 Video Non-activated HSC contraction induced by 10 nM ET-1; fluorescence latex beads (excited at 480 nm and emitted at 550 nm) move toward non-activated HSC just after treatment with 10 nM ET-1. Yellow circle indicates the non-activated HSC. The video plays at 330 × speed.
(MP4)

S1 Fig Measurement of reversibility in the contraction. (A) Effects of the Rho-kinase inhibitor H-1152 and the calmodulin inhibitor W-7 (G, H) on ET-1-induced contraction. Traces represent a typical time-course of changes in the length of bead movement after the treatment with H-1152 (1 μM) or W-7 (1 μM) on non-activated HSC contraction induced by ET-1 (10 nM). Traces in (A) were obtained. (B) Effects of NOC7, an NO donor, on ET-1-induced contraction. Traces represent typical time-courses of changes in the length of bead movement after treatment with NOC7 (100 μM) on non-activated HSC contraction induced by ET-1 (10 nM). (C) Bar graphs represent contraction after treatment with NOC7. Each bar represents the mean ± S.E.M. (n = 3).
(TIF)

S2 Fig Measurement of reference beads. The beads which are 400 μm distant from the cell are not affected by the cell contraction were used as reference beads.
(TIF)

S3 Fig RT-PCR analysis of the expression of cation channels in HSC. RT-PCR analysis of the expression of cation channels in non-activated and activated HSCs. The mRNA expression of T-, R-, L-, P/Q-type VDCC, STIM1, STIM2, Orai1, Orai2, Orai3, TRPC1, TRPC3, TRPC4, TRPC6, TRPV2, TRPV4, TRPM4, and TRPM7 was detected in both non-activated and activated HSCs.
(TIF)
