# Supplementary figures and images for: Quantitative real-time measurement of endothelin-1-induced contraction in single non-activated hepatic stellate cells
Source: PLoS One. 2021 Aug 3;16(8):e0255656. doi: 10.1371/journal.pone.0255656 (PMC8330899; doi:10.1371/journal.pone.0255656)

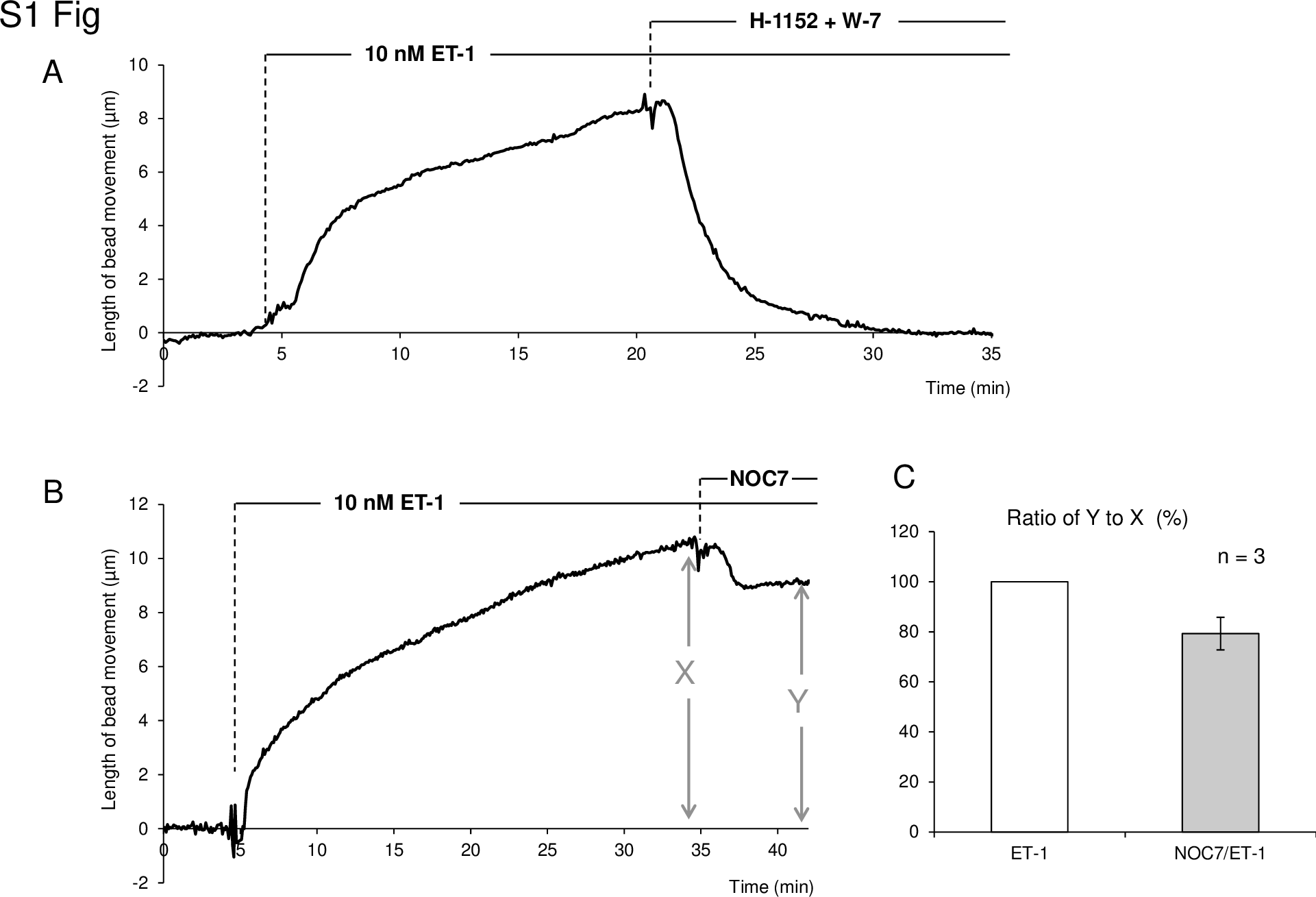

Supplement: S1 Fig — (A) Effects of the Rho-kinase inhibitor H-1152 and the calmodulin inhibitor W-7 (G, H) on ET-1-induced contraction. Traces represent a typical time-course of changes in the length of bead movement after the treatment with H-1152 (1 μM) or W-7 (1 μM) on non-activated HSC contraction induced by ET-1 (10 nM). Traces in (A) were obtained. (B) Effects of NOC7, an NO donor, on ET-1-induced contraction. Traces represent typical time-courses of changes in the length of bead movement after treatment with NOC7 (100 μM) on non-activated HSC contraction induced by ET-1 (10 nM). (C) Bar graphs represent contraction after treatment with NOC7. Each bar represents the mean ± S.E.M. (n = 3). (TIF) [file pone.0255656.s002.tif]

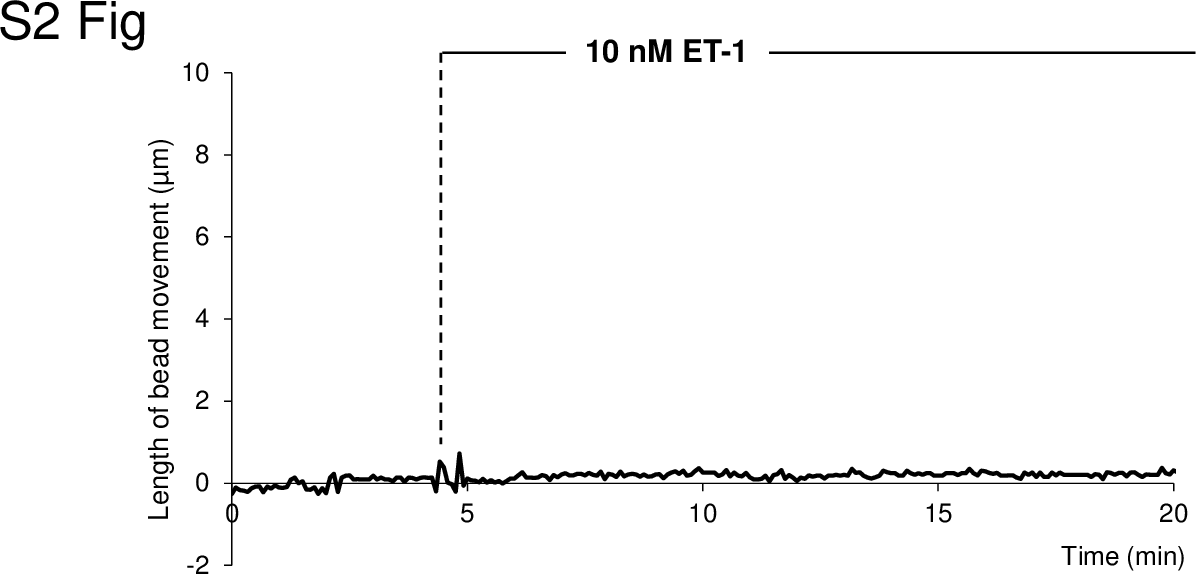

Supplement: S2 Fig — The beads which are 400 μm distant from the cell are not affected by the cell contraction were used as reference beads. (TIF) [file pone.0255656.s003.tif]

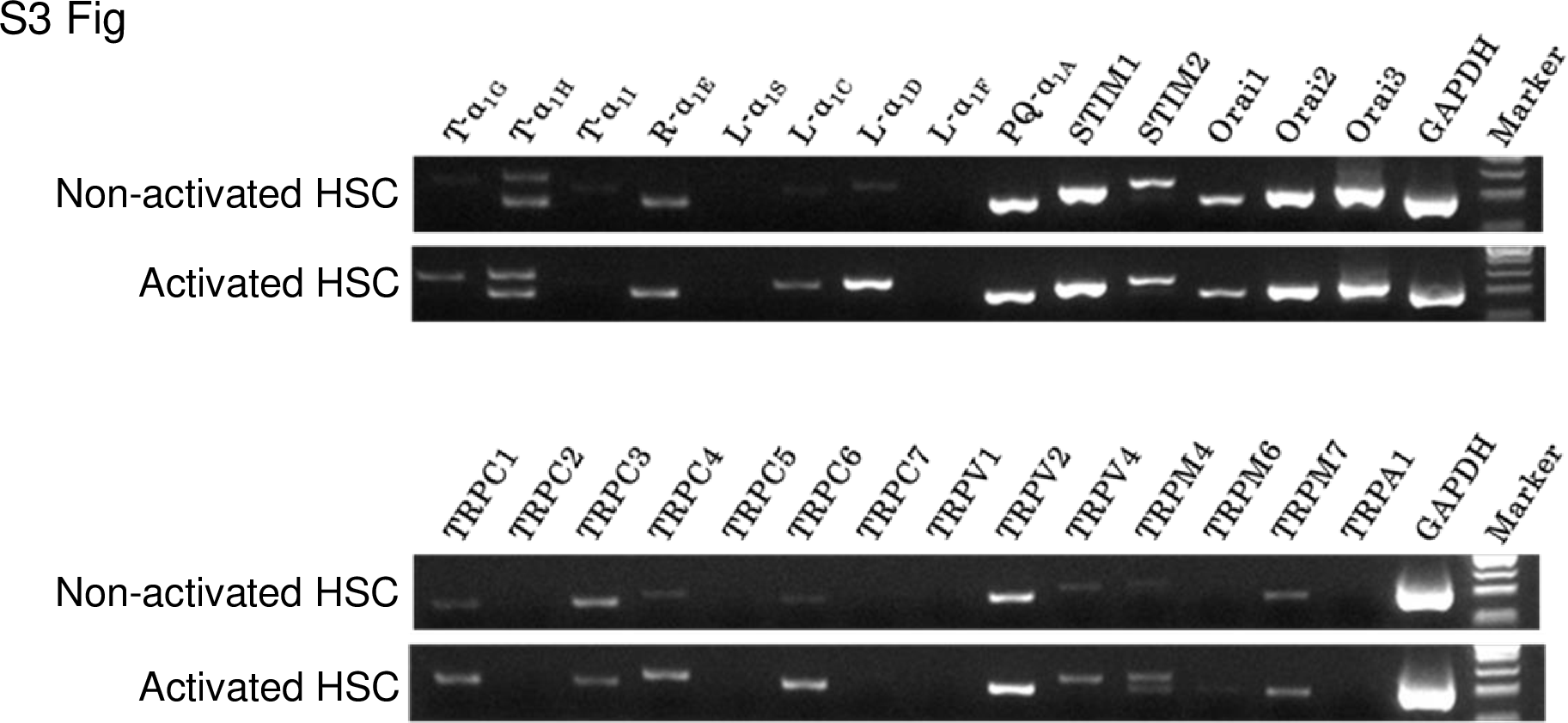

Supplement: S3 Fig — RT-PCR analysis of the expression of cation channels in non-activated and activated HSCs. The mRNA expression of T-, R-, L-, P/Q-type VDCC, STIM1, STIM2, Orai1, Orai2, Orai3, TRPC1, TRPC3, TRPC4, TRPC6, TRPV2, TRPV4, TRPM4, and TRPM7 was detected in both non-activated and activated HSCs. (TIF) [file pone.0255656.s004.tif]
